# Advancing radiation-induced mutant screening through high-throughput technology: a preliminary evaluation of mutant screening in *Arabidopsis thaliana*

**DOI:** 10.1186/s13007-025-01367-8

**Published:** 2025-04-15

**Authors:** Zhe Li, Jinhu Mu, Yan Du, Xiao Liu, Lixia Yu, Jianing Ding, Jing Long, Jingmin Chen, Libin Zhou

**Affiliations:** 1 Biophysics Group, Biomedical Center, Institute of Modern Physics, Chinese Academy of Sciences, Lanzhou, 730000 China; 2 University of Chinese Academy of Sciences, Beijing, 100049 China; 3 State Key Laboratory of Heavy Ion Science and Technology, Institute of Modern Physics, Chinese Academy of Sciences, Lanzhou, 730000 China

**Keywords:** *Arabidopsis thaliana*, Human-Machine recognition, Carbon ion beam, γ-Ray, High-throughput mutant screening

## Abstract

Identifying mutant traits is essential for improving crop yield, quality, and stress resistance in plant breeding. Historically, the efficiency of breeding has been constrained by throughput and accuracy. Recent significant advancements have been made through the development of automated, high-accuracy, and high-throughput equipment. However, challenges remain in the post-processing of large-scale image data and its practical application and evaluation in breeding. This study presents a comparative analysis of human and machine recognition, with validation of a randomly selected mutant at the physiological level performed on wild-type *Arabidopsis thaliana* and a candidate mutant of the M_3_ generation, which was generated through mutagenesis with heavy ion beams (HIBs) and ^60^Co-γ radiation. The mutant populations were subjected to image acquisition and automated screening using the High-throughput Plant Imaging System (HTPIS), generating approximately 10 GB of data (4,635 image datasets). We performed Principal Components Analysis (PCA), scatter matrix clustering, and Logistic Growth Curve (LGC) analyses, and compared these results with those obtained from traditional manual screening based on human visual assessment, and randomly selected #197 candidate mutants for validation in terms of growth and development, chlorophyll fluorescence, and subcellular structure. Our findings demonstrate that as the confidence interval level increases from 75 to 99.9%, the accuracy of machine-based mutant identification decreases from 1 to 0.446, while the false positive rate decreases from 0.817 to 0.118, and the false negative rate increases from 0 to 0.554. Nevertheless, machine-based screening remains more accurate and efficient than human assessment. This study evaluated and validated the efficiency (greater than 80%) of high-throughput techniques for screening mutants in complex populations of radiation-induced progeny, and presented a graphical data processing procedure for high-throughput screening of mutants, providing a basis for breeding techniques utilizing HIBs and γ-ray radiation, and offering innovative approaches and methodologies for radiation-induced breeding in the context of high-throughput big data.

**Supplementary Information:**

The online version contains supplementary material available at 10.1186/s13007-025-01367-8.

## Introduction

The advancement of crop breeding technologies is of utmost importance for global food production. It is estimated that by 2050, the world population will reach 9 to 10 billion, necessitating a 25–100% increase in food production to meet the escalating demand [1, 2]. However, the anticipated decline in crop yields and nutritional quality may result from the depletion of land resources, increasing pressure from pathogens and pests, and various biotic and abiotic stresses. In many regions, crop yields have plateaued, posing significant threats to global agricultural systems and food security [3, 4]. Therefore, enhancing the productivity of major global food crops relies heavily on crop varieties [5]. Although alternative methods such as de novo domestication and gene editing have been proposed to tackle these challenges, conventional breeding continues to be the predominant approach [6, 7]. While breeding accelerates the natural variation process in plants, efficiently identifying mutant lines with superior traits remains a significant bottleneck due to limitations in phenotypic analysis and screening systems [8].

To improve breeding efficiency, an increasing number of researchers are integrating radiation mutagenesis into their breeding strategies [9, 10]. Recently, HIBs have emerged as an effective physical mutagen, yielding significant results in breeding applications. HIBs have shown great potential in inducing genetic variation and improving crop traits. For example, Ishikawa et al. [11] obtained low cadmium-absorbing mutants from a M_2_ population of rice through HIBs mutagenesis, while Xiong et al. [12] constructed a high-capacity wheat mutant library that encompasses a broader range of genetic variations using high-energy carbon ion beams (CIBs). Additionally, Oliveira et al. [13] utilized multispectral and fluorescence technologies to reveal the stimulatory effects of different gamma radiation doses on soybeans. Ren et al. [14] conducted a systematic study on the mutagenic effects of various doses of CIBs across multiple generations of rice, evaluating genomic variant types and obtaining a significant number of valuable mutants. To date, the Mutant Variety Database of the Food and Agriculture Organization (FAO) and the International Atomic Energy Agency (IAEA) (http://mvd.IAEA.org) have documented more than 3,400 mutant varieties from various regions, including Asia, Europe, and North America. These varieties include major crops such as rice, wheat, soybean, and maize. Despite the advantages of HIBs in inducing mutations, efficiently identifying target mutants from the large number of mutated offspring remains a bottleneck in the breeding process.

Phenotype is a critical characteristic of organisms, located downstream of the “gene-transcript-protein-metabolism” cascade, and it directly reflects biological activities. Investigating phenotypes provides a deeper understanding of the “genotype-phenotype” relationship, facilitating the selection of optimal mutant offspring and accelerating crop genetic improvement [15]. Phenotypes, as observable traits of organisms, result from intricate interactions between genetic and environmental factors. This complexity significantly increases the difficulty of phenotypic screening in breeding programs. Traditional phenomics methods face several limitations, including high labor demands, time consumption, limited sample throughput, high costs, and frequent, invasive plant manipulations [16, 17]. These challenges hinder continuous, non-invasive monitoring throughout the plant life cycle, restricting the exploration of genetic resources and the breeding of important crop varieties. Consequently, they hinder progress in both plant breeding and functional genomics research [18].

With the rapid advancement of optical and sensor technologies, the availability of phenotyping equipment has surged, and high-throughput plant phenotyping tools have become widely adopted, revealing key traits associated with plant growth and development [18, 19]. These tools have been widely used in breeding research [20–23]. In contrast to conventional phenotyping techniques, high-throughput plant phenotyping tools integrate data acquisition devices, control terminals, and data analysis platforms. They facilitate non-invasive, fully automated collection and analysis of phenotypic data using imaging and spectroscopy technologies, capturing key traits such as dynamic growth, photosynthesis, and biomass within a short timeframe [24, 25]. These data can be used in combination with classical genetic analyses such as Quantitative Trait Locus (QTL), Genome-Wide Association Study (GWAS), and Phenome-wide association study (PheWAS) to identify genetic loci that are with greater functional significance [24, 26].

By incorporating high-throughput phenomics technology, researchers can efficiently screen for variants and process large sample datasets in a shorter time frame. This advancement enables breeders to quickly access the desired genetic resources and establishes a foundation for further investigation of functional genes and other critical information [27, 28]. Traditional screening methods primarily rely on manual identification, which is time-consuming and inefficient, especially when dealing with large populations [17]. This limitation makes it difficult to rapidly identify mutants with significant traits. Consequently, enhancing screening efficiency and accurately identifying mutants with breeding potential has become a key challenge in radiation mutagenesis breeding. While multifunctional, high-throughput platforms have advanced the field of phenotyping, comprehensive and reliable evaluations of their effectiveness in actual mutant screening, as well as the accuracy and integrity of the results, remain lacking. It is essential to develop thorough and effective analyses of the “big data” obtained from high-throughput phenomics platforms to extract valuable “biological information” that addresses these challenges [29, 30].

To explore this, we designed a comparative experiment to assess the effectiveness of mutant screening by manual versus machine recognition. In the experiment, we screened wild-type *Arabidopsis thaliana* and the M_3_ generation of candidate mutants induced by heavy ion beams (HIBs) and _60_Co-γ rays using both conventional visual recognition methods and HTPIS, and randomly selecting a candidate mutant for validation in terms of growth and development, chlorophyll fluorescence, and subcellular structure. The data obtained from HTPIS were subjected to Principal Components Analysis (PCA), scatter matrix clustering, and Logistic Growth Curve (LGC) analysis to compare the accuracy, false positive rate, and false negative rate of the two screening methods. By integrating available mutant information, the results of the HTPIS, and the validation data of candidate mutant strains, we aimed to propose a graphical data processing flow for high-throughput screening of radiation-induced progeny mutants.

## Materials and methods

### Plant materials and cultivation

The experimental materials included wild-type *Arabidopsis thaliana* (Columbia and *Landsberg erecta*) and M_3_ generation mutants with specific phenotypic variations induced by CIBs and ^60^Co-γ radiation. The ^60^Co-γ radiation source was provided by the Beijing Radiation Center, and the CIB was supplied by the Heavy Ion Research Facility in Lanzhou (31111.02.HIRFL). The energy and linear energy transfer (LET) of the CIB were 43.3 MeV/u and 50 keV/µm, respectively.

Wild-type and mutant seeds were sterilized with 10% sodium hypochlorite for 15 min, followed by rinsing with sterile water 3 to 8 times. The seeds were then evenly sown in pre-prepared Petri dishes containing Murashige and Skoog medium (0.8% agar, 1.5% sucrose) and labeled accordingly. After vernalization at 4 °C for 3 days, the seeds were transferred to a constant-temperature incubator at 23 °C for cultivation. Once the seedlings developed true leaves (approximately 8 days later), the mutant and wild-type plants were randomly transplanted into six-well plates designed for HTPIS detection, with specific positions recorded for each mutant. Two seedlings with the same identifier were transplanted in each well to ensure the survival of at least one plant, and excess seedlings were removed once stable growth was achieved stable growth. The number and proportion of mutants were independently determined by Group A (Table S1). All plants were grown under controlled conditions at 22 ± 1 °C, 60% relative humidity, with a light intensity of 108 µmol/m²/s, and an 18-hour light/6-hour dark photoperiod. Other conditions were consistent with those described by Du et al. [31].

### Experimental design and grouping

We established three experimental groups: Group A, Group B, and Group C. Group A is responsible for determining the types of mutants, the number of mutants to wild-type plants (Table S1, Table S2), and the planting positions of mutants on the HTS-specific six-well plates. This group will also compare and evaluate the experimental data and mutant screening results obtained from the different groups. Group B serves as the traditional screening group, employing manual phenotypic screening methods (i.e., human visual assessment) for mutant identification and result statistics. Group C functions as the high-throughput screening group, utilizing the high-throughput plant imaging system (Scanalyzer HTS, LemnaTec, Germany) for mutant identification and data analysis. Throughout the screening process, Groups A, B, and C operate independently, without any direct or indirect exchange or communication of experimental information.

### HTPIS mutant image acquisition and parameters

All plants in this study were imaged using the Scanalyzer HTS system, which operates in both visible light (VIS) and fluorescence imaging modes. The system automatically controls the movement of the camera, positioning it above the samples (such as multi-well plates or small pots) to capture images.

The visible light imaging mode allows for the measurement of over 50 phenotypic parameters, including plant structure, width, density, symmetry, leaf length, leaf width, leaf area, leaf angle, leaf color, leaf lesions, seed color, seed color area, and more. These phenotypic parameters are then represented as radar plots, which serve as a “fingerprint” of plant morphology. In this study, we primarily employed the LemnaGrid and Lemna Miner tools provided by Scanalyzer HTS for image integration and data preprocessing analysis.

### Phenotypic screening and data collection

Three to five days post-transplantation, Group B and C independently conducted mutant screening. The accuracy, false positive rate, and false negative rate of the screening results were statistically analyzed. Mutant identification was conducted using the HTPIS, and images of the plants were captured using the integrated visible light and fluorescence imaging systems. The accompanying analysis software (LemnaTec) extracted phenotypic data from the images.

The acquired phenotypic data underwent PCA for dimensionality reduction, and the resulting factor scores (Factor, FAC) as the screening data for the mutants. Subsequently, the mutant screening data were subjected to discrete matrix clustering analysis and LGC analysis. The details of the LGC model will be detailed as follows:

$$F=[\frac{h}{{1+{x_1} \times \exp ( - {x_2} \times {p_1})}} - k;\frac{H}{{1+{x_1} \times \exp ( - {x_2} \times {p_2})}} - s]$$

Notes: *h*,* H* were the maximum leaf areas at different time points (*p*_*1*_, *p*_*2*_), *x*_*1*_ and *x*_*2*_ were the fitting parameters and *k*,* s* were the correction values.

The integrated results from both phenotypic data analysis methods formed the final screening results for Group C. During the scatter matrix clustering analysis, confidence ellipse intervals were set at 95%, 97%, 99%, 99.5%, and 99.9%. These intervals were combined with the LGC analysis results to calculate the mutant screening accuracy, false positive rate, and false negative rate for each confidence level. Both screening methods were conducted every other day until the flowering stage of *Arabidopsis thaliana* was reached.

### Efficiency evaluation of the graphical data processing workflow for high-throughput screening of radiation-induced mutants in progeny

Within the screening framework, various mutant types and their corresponding positions were systematically identified. To evaluate the performance of the graphical data processing workflow, a comprehensive analysis was conducted. This involved performing PCA, scatter matrix clustering analysis, and LGC analysis on the data obtained from the HTPIS system. These analyses helped determine the number of mutants identified and facilitated the comparison of the accuracy, false positive rate, and false negative rate between the two screening methods. The accuracy was calculated as the proportion of correctly predicted mutants among all identified mutants:

$$\:\text{Accuracy=}\frac{\text{Correctly predicted mutants}}{\text{Total mutants }}$$

The false positive rate was determined as the proportion of incorrect predictions among all predicted mutants:

$$\:\text{False Positive Rate}=\frac{\text{Incorrectly predicted mutants}}{\text{Predicted mutants}}$$

The false negative rate was defined as the proportion of missed mutants among all actual mutants:

$$\:\text{False Negative Rate}=\frac{\text{Missed mutants}}{\text{Total mutants }}$$

To validate the graphical data processing workflow, one experimental repetition was conducted. The results obtained were compared against those from the traditional screening method to assess the accuracy and reliability of the graphical data processing workflow in identifying radiation-induced mutants.

### Determination of chlorophyll fluorescence parameters

The pulse-modulated fluorescence imaging system (Walz, Germany) was used for in vivo fluorescence measurement of *Arabidopsis thaliana* seedling leaves. Before measurement, the plants were dark-adapted for more than 30 minutes, and the parameters were set according to the instrument manual. Chlorophyll fluorescence kinetics curves and light response curves were measured, with specific indicators including Fv/Fm, which represents the actual photochemical efficiency of Photosystem II (PSII) under dark adaptation, ΦPSII, the actual photochemical efficiency of PSII, Non-Photochemical Quenching (NPQ), Electron Transport Rate (ETR) of PSII, and Photochemical Quenching (qP).

### Observation of leaf subcellular structure

The samples were cut into approximately 1 mm × 2 mm rectangular pieces and immediately placed into 2.5% glutaraldehyde fixative, ensuring complete immersion with the aid of a small amount of gauze, and fixed at 4 °C for at least 24 h. After fixation, the samples were washed three times with 0.2 M phosphate buffer (pH 7.4) for 15 min each time. The dehydration process was performed at room temperature, passing through a graded ethanol series (50%, 70%, 80%, 90%, 95%) for 15 min at each concentration, followed by treatment with absolute ethanol for 20 min, and immersion in pure acetone for 20 min. For infiltration, the samples were treated with a 2:1 mixture of anhydrous acetone and embedding resin at 37 °C for 1 h, followed by a 1:1 mixture of anhydrous acetone and embedding resin at 37 °C for 1 h, and then pure embedding resin at 37 °C overnight. After infiltration, the samples were carefully placed into embedding molds using a toothpick, and polymerization was allowed to proceed at 45 °C for 12 h. The embedded samples were sectioned into ultrathin sections using an ultramicrotome, stained with lead citrate for 15 min, washed three times with CO_2_-free ddH_2_O, stained with uranyl acetate for 15 min, washed again with ddH_2_O, and dried for observation under a transmission electron microscope (TEM-1230).

### Statistical analysis

Each treatment was conducted with three replicates, and data are presented as mean ± standard deviation (mean ± SD). ANOVA was performed using SPSS 27, followed by a post-hoc Tukey’s HSD test to determine significant differences (*P* < 0.05). Graphical representations were created using Origin 2019 and GraphPad Prism 9. Additionally, some data analysis and figures generation were carried out in R version 4.4.1 using the following packages: tidyverse version 2.0.0 [32], ggplot2 version 3.5.1, patchwork version 1.2.0, and RColorBrewer version 1.1-3.

## Results

### Traditional visual screening for mutants

Table 1 summarizes the accuracy, false positive rate, and false negative rate across the three experiments. In the first experiment, the accuracy was 0.875, with a false positive rate of 0.069 and a false negative rate of 0.125. The second experiment showed a drop in the accuracy to 0.429, with a false positive rate of 0.046 and a false negative rate of 0.571. The third experiment yielded an accuracy of 0.625, a false positive rate of 0.058, and a false negative rate of 0.375.

**Table 1:** The statistics results of three experiments used naked eye screening

Number of trials	1	2	3	Mean
No.of total plants	102	108	102	104
No. of mutants	8	14	8	10
The actual No. of mutants forecast	14	11	13	13
No.of mutants Correctly predicted	7	6	5	6
No.of mutants of prediction error	7	5	8	7
No.of mutants undetected	1	8	3	4
Accuracy	0.875	0.429	0.625	0.643
False positive rate	0.069	0.046	0.058	0.058
False negative rate	0.125	0.571	0.375	0.357

### HTPIS mutant image acquisition and phenotypic parameter PCA analysis

**Fig. 1:**
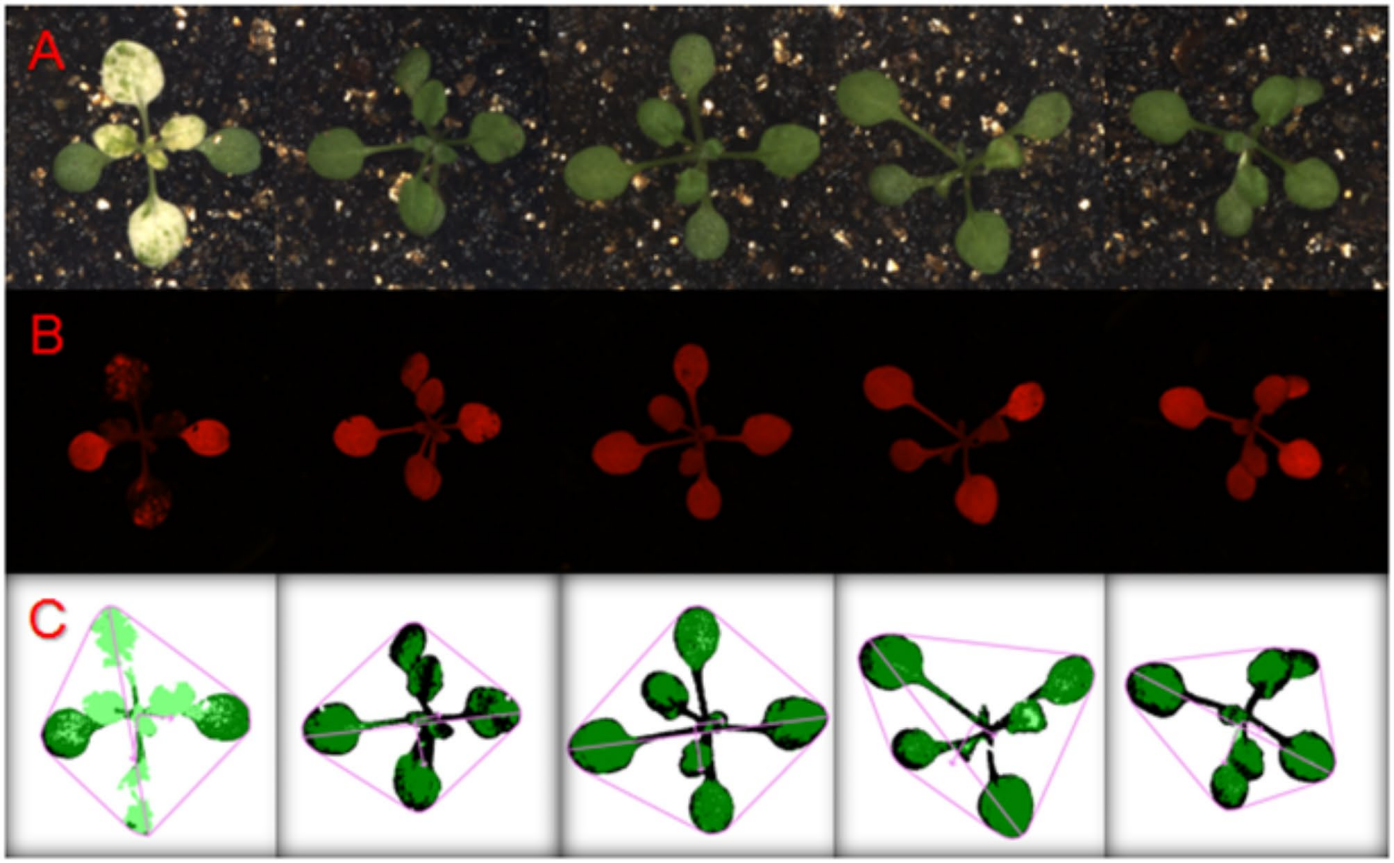
Plant phenotypic fingerprint image data obtained by HTPIS. (**A**) Visible light imaging; (**B**) Fluorescence imaging; (**C**) Phenotypic fingerprint map

**Fig. 2:**
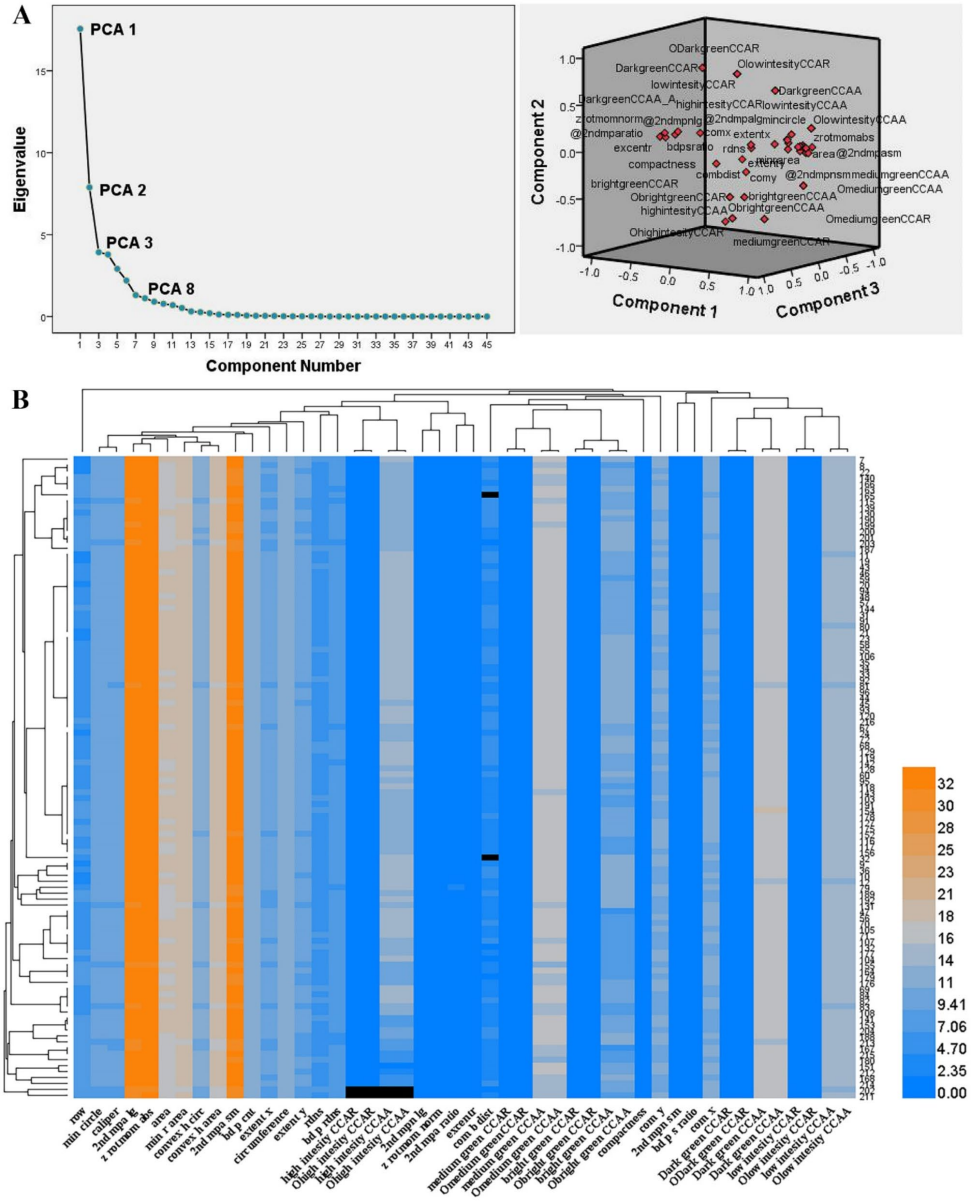
PCA analysis and cluster analysis results of machine recognition. (**A**) Principal component analysis. (PCA) and load map showing the distribution of phenotypic parameters and their contributions to the principal components. (**B**) Hierarchical cluster analysis of phenotypic parameters obtained using the High-Throughput Plant Imaging System (HTPIS). The X-axis represents the sample dendrogram generated by Ward’s method based on Euclidean distance, while the Y-axis corresponds to the unique identification numbers assigned to individual plants. The color scale indicates the extent of phenotypic variation, with darker shades reflecting greater differences

Using the HTPIS, images of hundreds of samples were collected and analyzed. Approximately 10 GB of plant phenotypic image data were obtained (Fig. 1), encompassing parameters such as leaf morphology, leaf color, fluorescence intensity, and area. PCA was conducted on 45 phenotypic parameters from the second screening results of the first experiment, using an eigenvalue threshold greater than 1. This analysis yielded 8 principal components along with their corresponding factor scores, with the variance contribution of these components exceeding 90% (Fig. 2A).

**Fig. 3:**
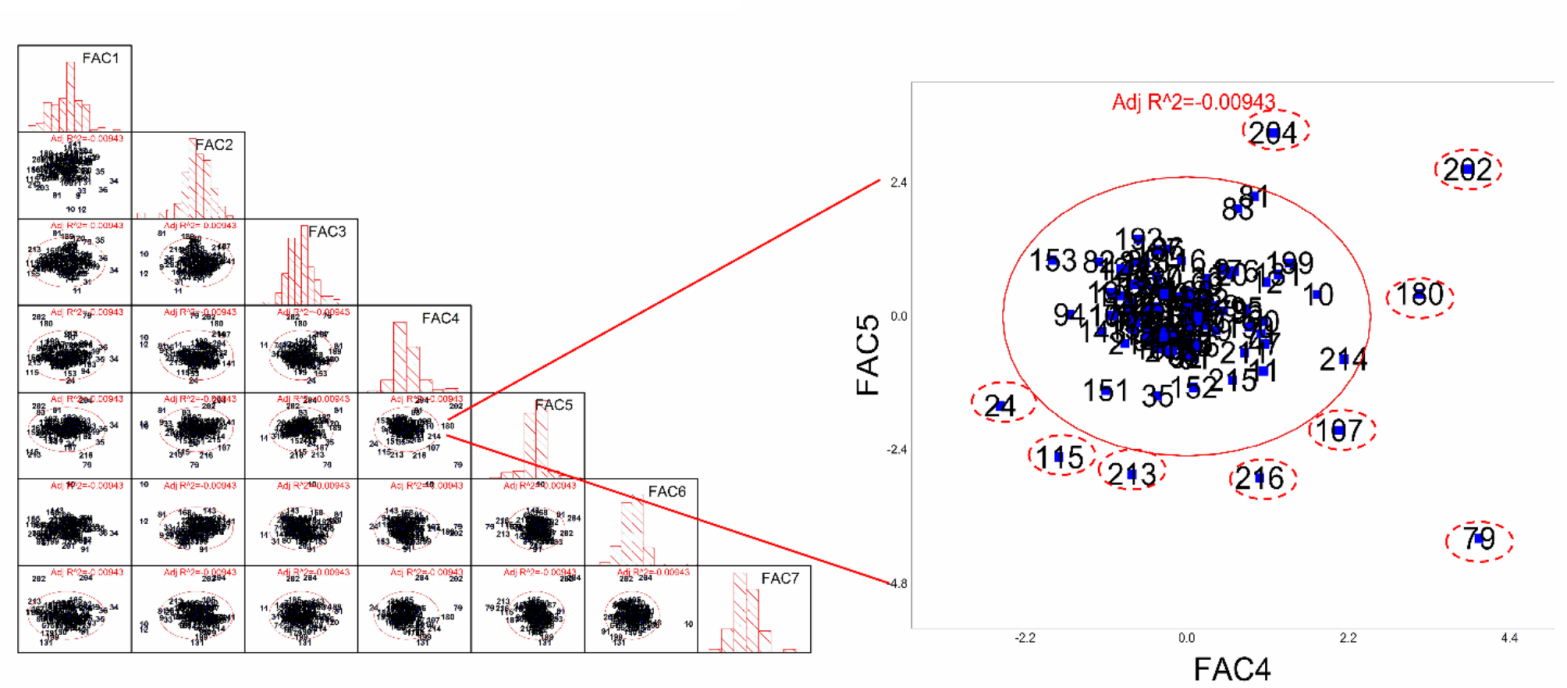
Scatter matrix cluster analysis. FAC1–FAC7 represents the principal component factors derived through dimensionality reduction using factor analysis, summarizing key phenotypic traits. Each point represents a plant and is labeled with a unique identifier. The solid red line highlights points outside the confidence interval ellipse (indicated by red dotted lines), which are identified as mutants

**Fig. 4:**
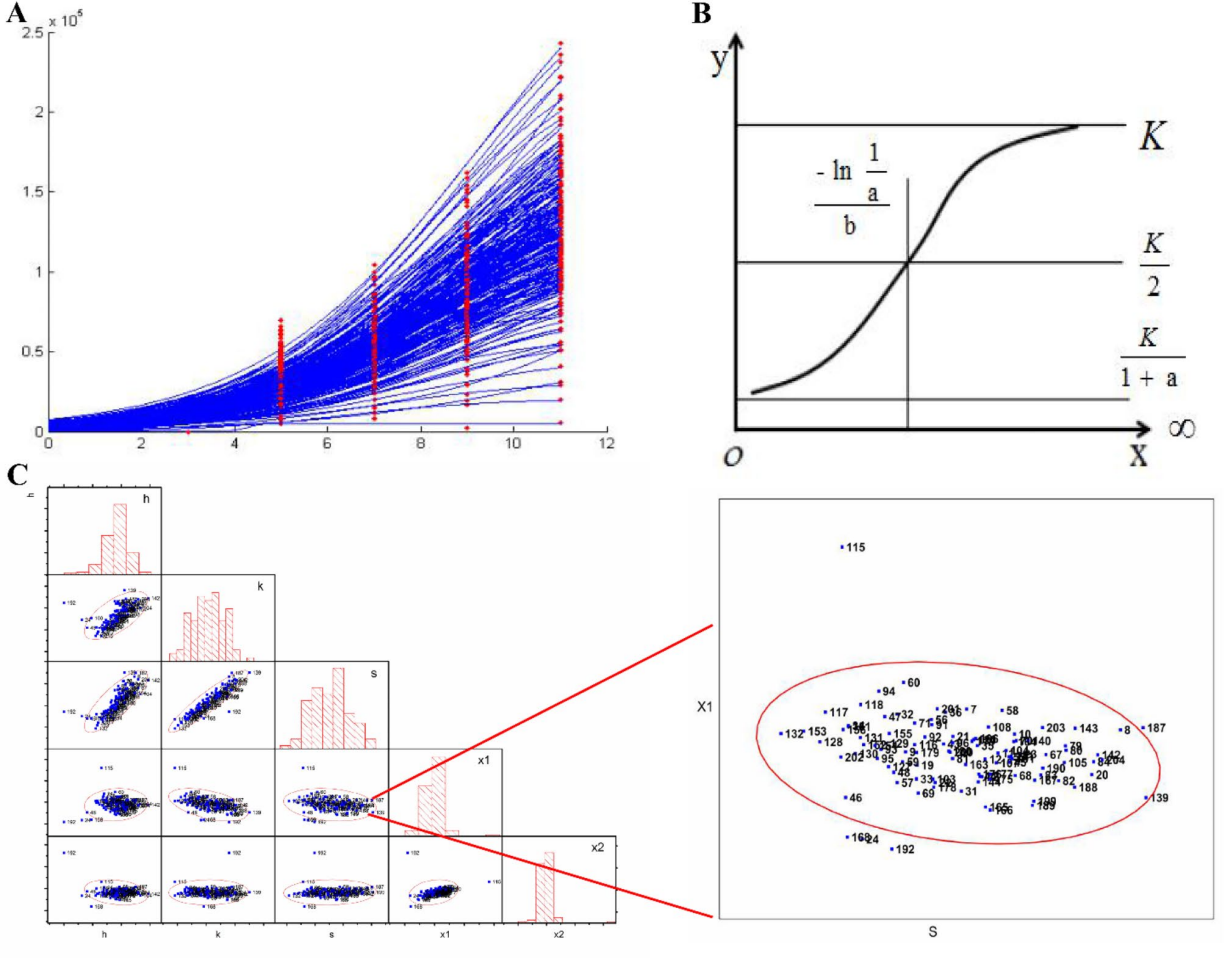
Leaf area growth curve parameter estimation and clustering analysis. (**A**) Schematic diagram showing the parameter values h, k, s, x_1_, and x_2_ derived from the LGC function model. (**B**) Schematic representation of the LGC function curve (By Chunxi Li et al. (“Biostatistics”)). (**C**) Pairwise clustering analysis was performed using the values of h, k, s, x_1_, and x_2_ obtained from each LGC function. The individuals outside the red circles are considered outliers and identified as candidate mutants

Considering that plants exhibit not only static phenotypic mutations based on physical characteristics but also dynamic growth features that change over time, we employed scatter matrix clustering analysis to evaluate the phenotypic changes. This approach facilitated mutant identification. Additionally, we created growth curves for leaf area measurements taken at different time points to discern differences in plant growth status among the mutants (Figs. 3 and 4).

The final results of the machine-based mutant screening combined the findings from scatter matrix clustering analysis and growth curve statistics. The clustering analysis of the phenotypic data indicated that most plants were successfully clustered, with only a few categories remaining unclustered, reflecting the proportions of wild types and mutants in the selected materials. The color differences in the visual representations highlight the phenotypic variations among the plants, with " black” colors corresponding to the planting numbers identified as mutants (Fig. 2B).

### Mutant screening with HTPIS at different confidence intervals

**Fig. 5:**
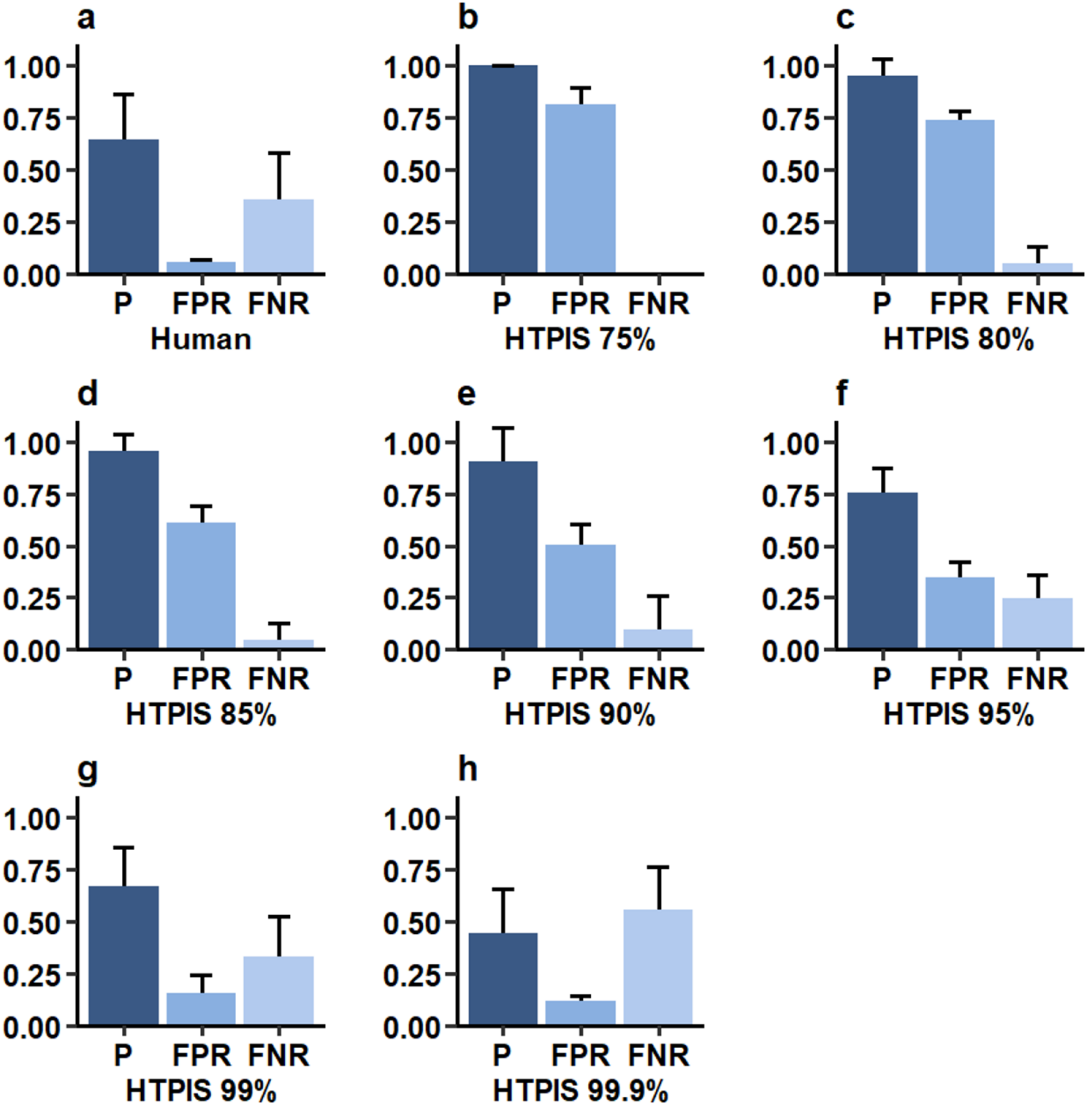
Performance comparison of traditional human eye recognition and HTPIS screening at different confidence intervals. The accuracy (P), false positive rate (FPR), and false negative rate (FNR) of traditional human eye recognition (**a**) and HTPIS screening at confidence intervals of 75% (**b**), 80% (**c**), 85% (**d**), 90% (**e**), 95% (**f**), 99% (**g**), and 99.9% (**h**)

To assess the accuracy of the HTPIS screening method, we averaged the results from three rounds of screening and calculated the accuracy (, P), false positive rate (FPR), and false negative rate (FNR) at different confidence intervals levels: 75%, 80%, 85%, 90%, 95%, 99%, and 99.9%. These results were then compared with those from traditional visual screening, as shown in Fig. 5.

Figure 5a displays the accuracy, false positive rate, and false negative rate for traditional visual screening. The accuracy of human visual assessment was 0.643, with a false positive rate of approximately 0.058 and a false negative rate of 0.357. The screening accuracy of HTPIS exhibited significant variations across the different confidence intervals.

At a confidence interval of 75% (Fig. 5b), the accuracy reached 1.0, while the false positive rate was notably high at 0.817. As the confidence interval increased, the false positive rate gradually decreased, while the false negative rate increased (Fig. 5b–h).

Specifically, at an 80% confidence interval (Fig. 5c), the accuracy was 0.952, with the false positive rate reduced to 0.742 and the false negative rate increasing to 0.082. At the highest confidence interval of 99.9% (Fig. 5h), the accuracy of HTPIS dropped to 0.446, while the false positive rate decreased to 0.118, and the false negative rate increased significantly to 0.554. In summary, both the screening accuracy and false positive rate of HTPIS markedly decrease as the confidence interval increases, while the false negative rate steadily rises. Therefore, selecting an appropriate confidence interval is crucial for achieving optimal screening outcomes in practical applications.

### Validation of graphical data processing workflows

Our study revealed significant differences in the accuracy of the two screening methods when the same mutant line was planted in different locations. Traditional visual screening often identified only a subset of the mutants or failed to recognize them all together, while the HTPIS successfully detected all mutants. Consequently, we conducted a verification experiment and randomly selected the #197 plant for verification at the physiological level.

#### Validation experiment results

**Fig. 6:**
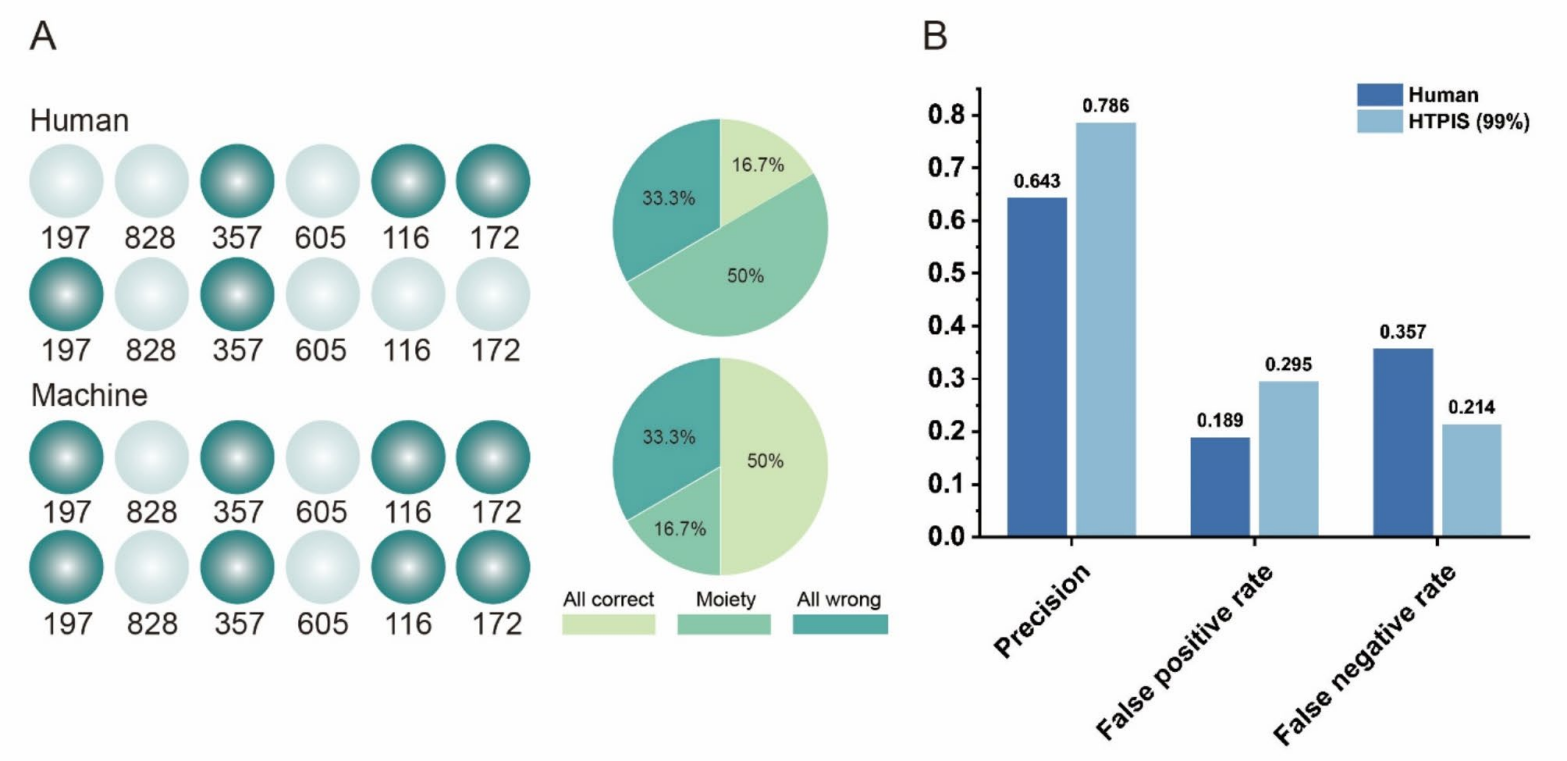
Validation experiment results (**A**). The results of the verification experiments from the man-machine comparison. The numerical values in the figure correspond to the names of the mutants; patterns represent different planting locations of the mutants. Dark patterns indicate that mutants at this location were correctly screened, while light patterns indicate that mutants at this location were not screened. The pie chart above shows the results of human-based screening, and the pie chart below shows the results of machine-based screening. (**B**). The results of accuracy, false positive, and false negative rates for the verification experiments in 99% confidence interval levels

The results indicated that the probability of HTPIS identifying all mutant lines was 50%, while traditional visual screening identified only 16.7 (Fig. 6A). Furthermore, the probability of both methods incorrectly identifying the same type of mutant planted in different positions was 33.3% (Fig. 6A). For the same type of mutant planted in various locations, traditional visual screening correctly identified mutants in half of the locations, yielding a probability of 50%, while HTPIS identified mutants in only 16.7%, This means that HTPIS accurately identified 83.3% of the positions (). At a 99% confidence interval, the accuracy of HTPIS screening was t 0.786, with a false positive rate of 0.295 and a false negative rate of 0.214 (Fig. 6B). These findings are consistent with previous experimental results (Fig. 5).

#### #197 Physiological Validation Results

**Table 2:** Effects of WT and #197 chlorophyll fluorescence parameters

	WT	#197
Fv/Fm	0.815 ± 0.010	0.796 ± 0.030**
Φ_PSII_	0.321 ± 0.010	0.198 ± 0.007**
NPQ	0.331 ± 0.008	0.312 ± 0.007
ETR	35.047 ± 1.222	21.022 ± 0.854**
qP	0.506 ± 0.014	0.332 ± 0.009*

**Fig. 7:**
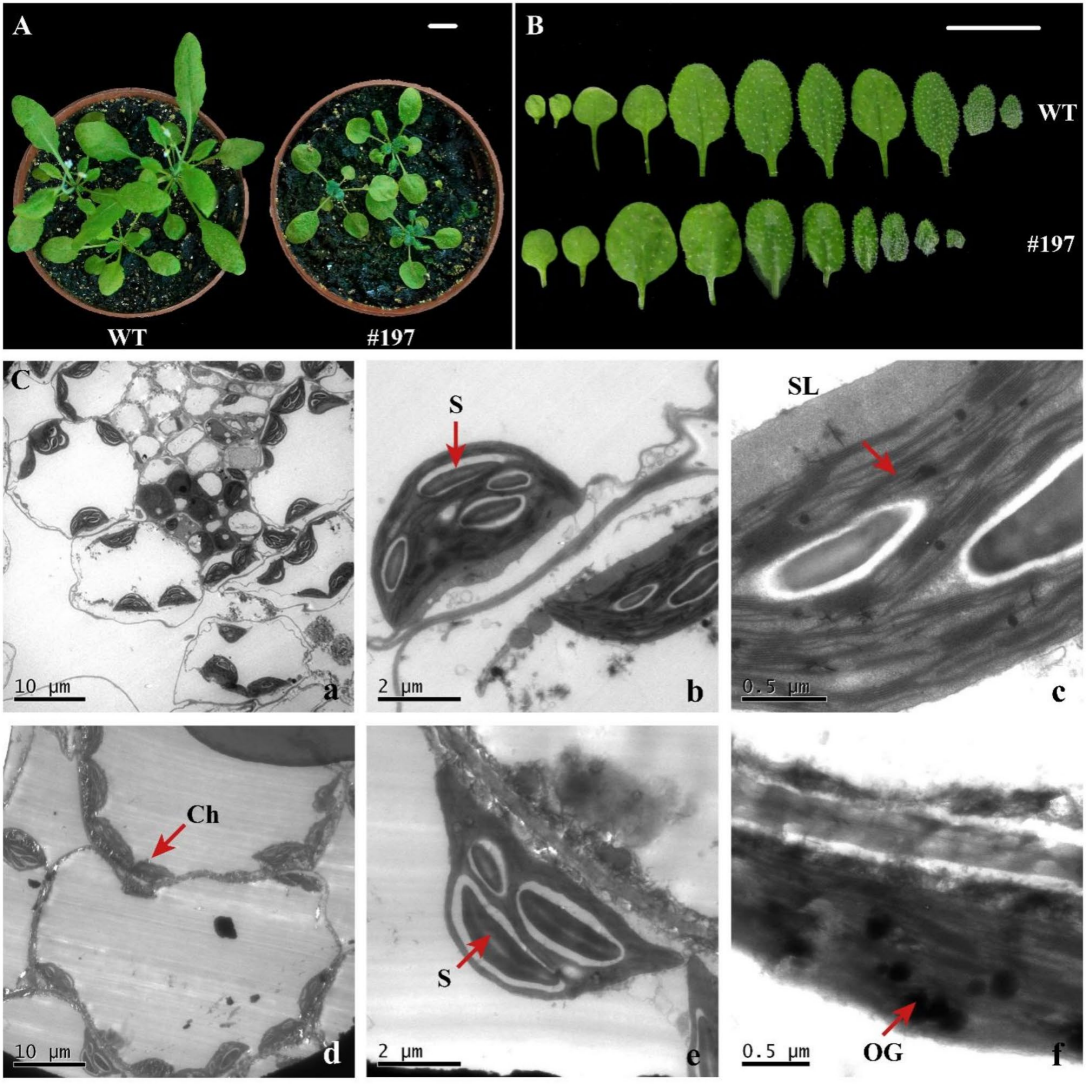
Leaf morphological phenotypic changes and third true leaf transmission electron microscopy observations of *Arabidopsis thaliana* #197 candidate mutants induced by carbon ion beam irradiation. (**A**) (**B**) Leaf morphological phenotypic changes (scale bar, 1 cm). (**C**) Third true leaf transmission electron microscopy. (**a**) WT-2500x. (**b**) WT-15,000x. (**c**) WT-60,000x. (**d**) 197-2500x. (**e**) 197 − 15,000x. (**f**) 197 − 50,000x. Notes: S, starch granule; SL, basal lamellae; Ch, chloroplast; OG, starved granule

During the growth stage, the #197 mutant exhibited frostbite-like symptoms, a pale green color, and thin young leaves, but these characteristics faded as the plant matured. The mature leaves became both concave and convex (Fig. 7A, B). Chlorophyll fluorescence measurements indicated that, compared to the wild type (WT), #197 showed reduced PSII activity, as evidenced by lower values of Fv/Fm, ΦPSII, ETR, and qP (Table 2). Additionally, in the rosette leaves, the central new leaves of #197 were dark green, thin, transparent, and appeared wilted. The first through fourth true leaves of #197 were larger than those of the wild type, while the mature rosette leaves display a rough, irregular surface.

To further investigate the subcellular structure of the different leaf types, transmission electron microscopy (TEM) was used. WT leaves at the same magnification revealed four large cells and numerous small cells (Fig. 7C-a), whereas #197 showed only one intact cell, with chloroplasts tightly adhered to the cell wall. The boundaries between adjacent chloroplasts were blurred, and the chloroplasts were more numerous (Fig. 7C-d). In individual chloroplasts, #197 contained 2–3 starch granules (Fig. 7C-e), while WT chloroplasts contained 4 starch granules (Fig. 7C-b). WT chloroplasts had neatly arranged grana thylakoids, with a few starch granules present in the stroma (Fig. 7C-c). In contrast, the grana thylakoids in #197 chloroplasts were disorganized, with some layers dissolved, and the starch granules were larger and densely distributed (Fig. 7C-f).

## Discussion

External environmental factors, along with internal gene expression, jointly determine plant growth throughout its life cycle [32]. Radiation can induce a diverse range of mutations in organisms, including insertions, deletions, inversions, and translocations in DNA [33]. Different types of radiation evoke varying biological effects, and due to their unique physical and biological characteristics, HIBs and other radiation types are widely utilized in plant breeding and radiation biology [34–36]. With the aid of high-throughput plant phenotyping tools, multidimensional phenotypic data can be rapidly acquired. When combined with the various types of mutants generated by heavy ion beam radiation, this approach effectively enhances the phenotypic identification of plant mutants, thereby advancing breeding research [14].

In this study, the accuracy of the three rounds of traditional visual screening ranged from a maximum of 0.875 to a minimum of 0.429 (Table 1). Although the false positive rate was low, the high false negative rate indicates that some mutants were missed during traditional visual screening. The results from the second experimental screening differed significantly from those of the first and third rounds. Upon analysis, we found that in the second repeat experiment, members of Group A had performed repeated plantings of the same mutant, resulting in fewer mutant types and an increased total number of mutant plants. Interestingly, when the same type of mutant was planted in different locations, traditional visual screening typically only identified one or few correct positions. This suggests that traditional visual screening is influenced by subjective factors, which likely contributed to the lower accuracy and higher false negative rate observed in the second round of experiments.

When using HTPIS for screening, as the confidence interval gradually increases (from 95 to 99.9%), we find that the traditional human eye recognition screening has a certain degree of accuracy, with a gradual decrease in the false positive rate and a gradual increase in the false negative rate. Within the 75% confidence interval, the false-negative rate of HTPIS screening is 0. As the confidence interval increases from 75 to 99.9%, the accuracy of machine screening gradually decreases, but within the 99% confidence interval, the accuracy of HTPIS screening is much higher than that of traditional human eye recognition screening; the confidence interval increases from 75 to 99.9%. The false positive rates of HTPIS screening were higher than those of traditional human eye screening, and the false positive rates of machine screening gradually increased as the confidence interval increased; the false negative rates of machine screening gradually increased as the confidence interval increased from 75 to 99.9%. The accuracy of machine screening was higher than that of traditional human eye screening, and the false-negative rate was lower than that of traditional human eye screening within the 99% confidence interval (Fig. 5a–h). In addition, the results of growth and development, chlorophyll fluorescence, and subcellular structure of candidate mutant #197 indicate that HTPIS can more accurately screen phenotypic mutants with subtle changes (Fig. 7, Fig. S1). These findings indicate that compared to traditional visual screening, HTPIS screening is less likely to overlook mutants. Therefore, it can effectively replace traditional visual methods for large-scale mutant screening.

When the same mutant line is planted in different locations, significant discrepancies in accuracy between the two screening methods are observed. Traditional screening methods often identify only a subset of mutants or may incorrectly assess all planting positions. This limitation primarily stems from the reliance on the observer’s visual judgment, which can be influenced by subjective factors such as experience, fatigue, and distractions. Additionally, environmental variations at different planting locations (e.g., light and soil conditions) may affect the phenotypic expression of the plants, causing some mutants to be less prominent in specific locations, which can result in their omission or misidentification. In contrast, when used for screening, all types of mutants can be accurately identified regardless of their planting location. This demonstrates that, compared to the somewhat subjective traditional visual screening method, it offers greater accuracy, higher efficiency in mutant identification, and a stronger ability to avoid overlooking mutants.

While using HTPIS for mutant screening has improved accuracy to some extent, its false positive rate remains significantly higher than that of traditional visual screening. Notably, an increase in the false positive rate does not necessarily indicate a decrease in screening effectiveness. This phenomenon primarily reflects the sensitivity of HTS technology in detecting macro-level phenotypic changes, enabling HTPIS to capture subtle micro-phenotypic variations that are difficult to discern with the human eyes. Consequently, even with false positives, it provides a richer array of potential mutant information, laying a solid foundation for subsequent screening and research. From a high-throughput perspective, employing it can conserve resources while maintaining accuracy. Integrating traditional visual screening with the platform in crop breeding and plant mutant research has the potential to significantly enhance mutant screening efficiency. Specifically, the mutant population can first undergo initial screening using HTPIS, followed by traditional visual screening to refine the selection by eliminating a large number of non-mutants. This approach not only helps reduce resource consumption but also accelerates the acquisition of desired mutant plants, thereby improving overall research efficiency.

Mutant screening is a crucial aspect of breeding, and high-throughput technologies, along with their associated screening methods, show great promise in identifying mutants and domesticating wild resources. High-throughput screening techniques allow for the identification of mutants with significant agronomic traits at early seedling stages for further analysis. This early screening capability is crucial for accelerating mutant identification and the breeding processes [37]. As these technologies rapidly develop, they will provide more precise and efficient tools for plant breeding and genetic improvement. For instance, Ma et al. [38] reported a portable phenotyping device for seed morphological parameters based on smartphone technology, providing a convenient means for phenotypic data collection. Wang et al. [39] utilized a fully automated 3D high-throughput phenotyping platform to monitor maize inbred lines throughout their entire growth period. They employed a batch processing program for multi-optical images to analyze and extract phenotypic traits (i-traits), which, combined with genome-wide association studies (GWAS), identified core SNPs and candidate genes related to specific phenotypes, constructing a gene-phenotype association network to deeply explore the dynamic genetic basis and regulatory networks of maize plant height. Additionally, Tang et al. [40] developed an image-based strategy for phenotypic acquisition and analysis in rice, providing a novel approach for the extraction and analysis of phenotypes throughout the entire growth period. Similarly, Kurbanov et al. [41] assessed the field germination rates of soybean crops using multispectral data (MSD), demonstrating the potential of high-throughput technologies for agricultural applications. Therefore, combining high-throughput phenomics technologies with ion beam mutagenesis breeding represents a significant advancement in modern plant breeding. By integrating these advanced technologies, we can not only enhance mutant screening efficiency but also provide more reliable scientific evidence for varietal improvement.

While visible light has been widely used for screening and identifying phenotypic traits such as leaf structure and plant morphology, the introduction of infrared and fluorescence spectroscopy offers new possibilities for more in-depth analysis of physiological and biochemical characteristics [42, 43]. Infrared spectroscopy effectively detects water status and chlorophyll content in plants, while fluorescence spectroscopy is useful for detecting metabolic changes and stress responses within the plant [44, 45]. Integrating these technologies will further enrich the diversity of phenotypic data and enhance the accuracy and reliability of mutant screening. In this study, we compared the accuracy, false positive rate, and false negative rate of HTPIS and traditional visual screening methods. With approximately 10 GB of image data including 45 phenotypic parameters from 4,635 images in a screening population comprising just about 300 individuals, as the population expands, this data will surge accordingly. During breeding, the screening population often reaches tens of thousands of individuals, therefore, storing, retrieving, and interpreting this massive amount of data to swiftly obtain screening results presents a formidable challenge. Recent advancements, such as the development of the DSConv-GAN model for disease detection in Chinese cabbage using drones and deep learning, the YOLO-WDNet network for cotton and weed detection, and deep learning-based high-accuracy soybean phenotype extraction, suggest that similar technologies could be applied to radiation-induced mutant screening [46–48]. Based on these research developments, we speculate that the application of deep learning, convolutional neural networks (CNNs), artificial intelligence, and cloud computing technologies in radiation-induced mutant screening may significantly enhance the efficiency of processing large-scale phenotypic data. The integration of these technologies can not only reduce equipment costs but also enable real-time data collection and processing in various environmental conditions (such as in the field), allowing for the detection of subtle variations in plant growth. This will provide more efficient automated tools for plant breeding and mutant screening, further advancing plant science research and agricultural production. However, despite the great potential of these technologies, their application in radiation-induced mutant screening still requires further validation and experimental research.

## Conclusion

This study introduces a foundational high-throughput graphical data processing workflow specifically designed for screening radiation-induced progeny mutants. By systematically applying high-throughput technology to radiation mutagenesis breeding, we demonstrate its potential to significantly improve the efficiency of identifying radiation-induced mutant materials. This workflow enables researchers to efficiently analyze large-scale image datasets generated by high-throughput devices, facilitating the accurate selection of candidate mutants. While HTPIS offers substantial improvements in screening efficiency (by more than 80%) compared to traditional visual screening, which is prone to subjectivity and a high false negative rate, its relatively high false positive rate limits its ability to fully replace traditional methods. We therefore recommend using HTPIS as an initial screening tool, followed by manual validation, which combines the strengths of both methods and improves overall accuracy and reliability in mutant identification. Since HTPIS equipment is primarily suited for screening phenotypic variations in small plants and seedlings, we speculate that combining new algorithms or detection technologies may offer new directions for large-scale mutant screening. While these technologies show potential in plant phenotypic analysis, their application in radiation-induced mutant screening still requires further validation and research.

## Electronic supplementary material

Below is the link to the electronic supplementary material.

Supplementary Material 1

Supplementary Material 2

## Data Availability

No datasets were generated or analysed during the current study.

## References

[1] Hunter MC, Smith RG, Schipanski ME, Atwood LW, Mortensen DA. Agriculture in 2050: recalibrating targets for sustainable intensification. Bioscience. 2017;67(4):386–91. 10.1093/biosci/bix010.

[2] Tilman D, Balzer C, Hill J, Befort BL. Global food demand and the sustainable intensification of agriculture. 2011, 108(50):20260–4. 10.1073/pnas.1116437108 10.1073/pnas.1116437108 PMC3250154 22106295

[3] Steinwand MA, Ronald PC. Crop biotechnology and the future of food. Nat Food. 2020;1(5):273–83. 10.1038/s43016-020-0072-3.

[4] Strange RN, Scott PR. Plant disease: a threat to global food security. Annu Rev Phytopathol. 2005;43:83–116. 10.1146/annurev.phyto.43.113004.133839. 16078878 10.1146/annurev.phyto.43.113004.133839

[5] Bailey-Serres J, Parker JE, Ainsworth EA, Oldroyd GED, Schroeder JI. Genetic strategies for improving crop yields. Nature. 2019;575(7781):109–18. 10.1038/s41586-019-1679-0. 31695205 10.1038/s41586-019-1679-0 PMC7024682

[6] Liu J, Fernie AR, Yan J. Crop breeding – From experience-based selection to precision design. J Plant Physiol. 2021;256:153313. 10.1016/j.jplph.2020.153313. 33202375 10.1016/j.jplph.2020.153313

[7] Yu H, Lin T, Meng X, Du H, Zhang J, Liu G, Chen M, Jing Y, Kou L, Li X, et al. A route to de Novo domestication of wild allotetraploid rice. Cell. 2021;184(5):1156–e11701114. 10.1016/j.cell.2021.01.013. 33539781 10.1016/j.cell.2021.01.013

[8] Islam NU, Wani SH, Ali G, Dar ZA, Wani A, Lone A. High-throughput phenotyping for abiotic stress resilience in cereals. J Cereal Res 2021, 13(1).

[9] Stadler LJ. Mutations in barley induced by X-Rays and radium. Science. 1928;68(1756):186–7. 10.1126/science.68.1756.186. 17774921 10.1126/science.68.1756.186

[10] Muller HJ. The Production of Mutations by X-Rays1. *Proceedings of the National Academy of Sciences* 1928, 14(9):714–726. 10.1073/pnas.14.9.714 10.1073/pnas.14.9.714 PMC1085688 16587397

[11] Ishikawa S, Ishimaru Y, Igura M, Kuramata M, Abe T, Senoura T, Hase Y, Arao T, Nishizawa NK, Nakanishi H. Ion-beam irradiation, gene identification, and marker-assisted breeding in the development of low-cadmium rice. Proc Natl Acad Sci U S A. 2012;109(47):19166–71. 10.1073/pnas.1211132109. 23132948 10.1073/pnas.1211132109 PMC3511095

[12] Xiong H, Guo H, Fu M, Xie Y, Zhao L, Gu J, Zhao S, Ding Y, Du Q, Zhang J, et al. A large-scale whole-exome sequencing mutant resource for functional genomics in wheat. Plant Biotechnol J. 2023;21(10):2047–56. 10.1111/pbi.14111. 37401008 10.1111/pbi.14111 PMC10502753

[13] Oliveira NM, Medeiros ADd, Nogueira ML, Arthur V, Mastrangelo TA, Barboza da Silva C. Hormetic effects of low-dose gamma rays in soybean seeds and seedlings: A detection technique using optical sensors. Comput Electron Agric. 2021;187:106251. 10.1016/j.compag.2021.106251.

[14] Ren W, Wang H, Du Y, Li Y, Feng Z, Zhou X, Kang G, Shu Q, Guo T, Guo H, et al. Multi-generation study of heavy ion beam-induced mutations and agronomic trait variations to accelerate rice breeding. Front Plant Sci. 2023;14:1213807. 10.3389/fpls.2023.1213807. 37416884 10.3389/fpls.2023.1213807 PMC10322207

[15] Houle D, Govindaraju DR, Omholt S. Phenomics: the next challenge. Nat Rev Genet. 2010;11(12):855–66. 10.1038/nrg2897. 21085204 10.1038/nrg2897

[16] Chen D, Neumann K, Friedel S, Kilian B, Chen M, Altmann T, Klukas C. Dissecting the phenotypic components of crop plant growth and drought responses based on high-throughput image analysis. Plant Cell. 2014;26(12):4636–55. 25501589 10.1105/tpc.114.129601 PMC4311194

[17] Furbank RT, Tester M. Phenomics–technologies to relieve the phenotyping bottleneck. Trends Plant Sci. 2011;16(12):635–44. 22074787 10.1016/j.tplants.2011.09.005

[18] Campbell ZC, Acosta-Gamboa LM, Nepal N, Lorence A. Engineering plants for tomorrow: how high-throughput phenotyping is contributing to the development of better crops. Phytochem Rev. 2018;17(6):1329–43. 10.1007/s11101-018-9585-x.

[19] Jangra S, Chaudhary V, Yadav RC, Yadav NR. High-throughput phenotyping: A platform to accelerate crop improvement. Phenomics. 2021;1(2):31–53. 10.1007/s43657-020-00007-6 36939738 10.1007/s43657-020-00007-6 PMC9590473

[20] Santana DC, de Oliveira Cunha MP, dos Santos RG, Cotrim MF, Teodoro LPR, da Silva Junior CA, Baio FHR, Teodoro PE. High-throughput phenotyping allows the selection of soybean genotypes for earliness and high grain yield. Plant Methods. 2022;18(1):13. 10.1186/s13007-022-00848-4. 35109882 10.1186/s13007-022-00848-4 PMC8812231

[21] Wu X, Feng H, Wu D, Yan S, Zhang P, Wang W, Zhang J, Ye J, Dai G, Fan Y, et al. Using high-throughput multiple optical phenotyping to decipher the genetic architecture of maize drought tolerance. Genome Biol. 2021;22(1):185. 10.1186/s13059-021-02377-0 34162419 10.1186/s13059-021-02377-0 PMC8223302

[22] Wang C, Caragea D, Kodadinne Narayana N, Hein NT, Bheemanahalli R, Somayanda IM, Jagadish SVK. Deep learning based high-throughput phenotyping of chalkiness in rice exposed to high night temperature. Plant Methods. 2022;18(1):9. 10.1186/s13007-022-00839-5. 35065667 10.1186/s13007-022-00839-5 PMC8783510

[23] Resende EL, Bruzi AT, Cardoso ES, Carneiro VQ, Pereira de Souza VA. Frois Correa Barros PH, Pereira RR: High-throughput phenotyping: application in maize breeding. AgriEngineering. 2024;6(2):1078–92.

[24] Mir RR, Reynolds M, Pinto F, Khan MA, Bhat MA. High-throughput phenotyping for crop improvement in the genomics era. Plant Sci. 2019;282:60–72. 10.1016/j.plantsci.2019.01.007. 31003612 10.1016/j.plantsci.2019.01.007

[25] Neilson EH, Edwards AM, Blomstedt CK, Berger B, Møller BL, Gleadow RM. Utilization of a high-throughput shoot imaging system to examine the dynamic phenotypic responses of a C4 cereal crop plant to nitrogen and water deficiency over time. J Exp Bot. 2015;66(7):1817–32. 10.1093/jxb/eru526. 25697789 10.1093/jxb/eru526 PMC4378625

[26] Yang W, Zhang X, Duan L. High-throughput Phenotyping (HTP) and Genetic Analysis Technologies Reveal the Genetic Architecture of Grain Crops. In: *High-Throughput Crop Phenotyping.* Edited by Zhou J, Nguyen HT. Cham: Springer International Publishing; 2021: 101–127.

[27] Knoch D, Abbadi A, Grandke F, Meyer RC, Samans B, Werner CR, Snowdon RJ, Altmann T. Strong Temporal dynamics of QTL action on plant growth progression revealed through high-throughput phenotyping in Canola. Plant Biotechnol J. 2020;18(1):68–82. 10.1111/pbi.13171. 31125482 10.1111/pbi.13171 PMC6920335

[28] Meyer RC, Weigelt-Fischer K, Knoch D, Heuermann M, Zhao Y, Altmann T. Temporal dynamics of QTL effects on vegetative growth in *Arabidopsis thaliana*. J Exp Bot. 2020;72(2):476–90. 10.1093/jxb/eraa490 10.1093/jxb/eraa490 33080013

[29] Reynolds D, Ball J, Bauer A, Davey R, Griffiths S, Zhou J. CropSight: a scalable and open-source information management system for distributed plant phenotyping and IoT-based crop management. Gigascience. 2019;8(3):giz009. 30715329 10.1093/gigascience/giz009 PMC6423370

[30] Gill T, Gill SK, Saini DK, Chopra Y, de Koff JP, Sandhu KS. A comprehensive review of high throughput phenotyping and machine learning for plant stress phenotyping. Phenomics. 2022;2(3):156–83. 10.1007/s43657-022-00048-z. 36939773 10.1007/s43657-022-00048-z PMC9590503

[31] Du Y, Feng Z, Wang J, Jin W, Wang Z, Guo T, et al. Frequency and spectrum of mutations induced by gamma rays revealed by phenotype screening and Whole-Genome Re-Sequencing in *Arabidopsis thaliana*. Int J Mol Sci. 2022;23(2). 10.3390/ijms23020654 10.3390/ijms23020654 PMC8775868 35054839

[32] Volkov AG, Ranatunga DRA. Plants as environmental biosensors. Plant Signal Behav. 2006;1(3):105–15. 10.4161/psb.1.3.3000. 19521490 10.4161/psb.1.3.3000 PMC2635006

[33] Tanaka A, Shikazono N, Hase Y. Studies on biological effects of ion beams on lethality, molecular nature of mutation, mutation rate, and spectrum of mutation phenotype for mutation breeding in higher plants. J Radiat Res. 2010;51(3):223–33. 10.1269/jrr.09143. J Journal of Radiation Research. 20505261 10.1269/jrr.09143

[34] Zhang X, Yang F, Ma H, Li J. Evaluation of the Saline-Alkaline tolerance of rice (Oryza sativa L.) mutants induced by Heavy-Ion beam mutagenesis. Biology (Basel). 2022;11(1). 10.3390/biology11010126. 10.3390/biology11010126 PMC8773086 35053124

[35] Du Y, Luo S, Zhao J, Feng Z, Chen X, Ren W, et al. Genome and transcriptome-based characterization of high energy carbon-ion beam irradiation induced delayed flower senescence mutant in *Lotus japonicus*. BMC Plant Biol. 2021;21(1):510. 10.1186/s12870-021-03283-0 34732128 10.1186/s12870-021-03283-0 PMC8564971

[36] Hase Y, Okamura M, Takeshita D, Narumi I, Tanaka A. Efficient induction of flower-color mutants by ion beam irradiation in Petunia seedlings treated with high sucrose concentration. Plant Biotechnol. 2010;27(1):99–103.

[37] Maggiorelli A, Baig N, Prigge V, Bruckmuller J, Stich B. Using drone-retrieved multispectral data for phenomic selection in potato breeding. Theor Appl Genet. 2024;137(3):70. 10.1007/s00122-024-04567-3. 38446220 10.1007/s00122-024-04567-3 PMC10917832

[38] Zhihong M, Yuhan M, Liang G, Chengliang L. Smartphone-Based visual measurement and portable instrumentation for crop seed phenotyping. IFAC-PapersOnLine. 2016;49(16):259–64. 10.1016/j.ifacol.2016.10.048.

[39] Wang W, Guo W, Le L, Yu J, Wu Y, Li D, Wang Y, Wang H, Lu X, Qiao H, et al. Integration of high-throughput phenotyping, GWAS, and predictive models reveals the genetic architecture of plant height in maize. Mol Plant. 2023;16(2):354–73. 10.1016/j.molp.2022.11.016. 36447436 10.1016/j.molp.2022.11.016 PMC11801313

[40] Tang Z, Chen Z, Gao Y, Xue R, Geng Z, Bu Q, Wang Y, Chen X, Jiang Y, Chen F, et al. A strategy for the acquisition and analysis of Image-Based phenome in rice during the whole growth period. Plant Phenomics. 2023;5:0058. 10.34133/plantphenomics.0058. 37304154 10.34133/plantphenomics.0058 PMC10249964

[41] Kurbanov R, Panarina V, Polukhin A, Lobachevsky Y, Zakharova N, Litvinov M, Rebouh NY, Kucher DE, Gureeva E, Golovina E, et al. Evaluation of field germination of soybean breeding crops using multispectral data from UAV. Agronomy. 2023;13(5):1348.

[42] Buitrago MF, Skidmore AK, Groen TA, Hecker CA. Connecting infrared spectra with plant traits to identify species. ISPRS J Photogrammetry Remote Sens. 2018;139:183–200. 10.1016/j.isprsjprs.2018.03.013.

[43] Ribeiro da Luz B. Attenuated total reflectance spectroscopy of plant leaves: a tool for ecological and botanical studies. New Phytol. 2006;172(2):305–18. 16995918 10.1111/j.1469-8137.2006.01823.x

[44] Maimaitiyiming M, Ghulam A, Bozzolo A, Wilkins JL, Kwasniewski MT. Early detection of plant physiological responses to different levels of water stress using reflectance spectroscopy. Remote Sens. 2017;9(7):745.

[45] Liu W, Li Y, Tomasetto F, Yan W, Tan Z, Liu J, et al. Non-destructive measurements of *Toona sinensis* chlorophyll and nitrogen content under drought stress using near infrared spectroscopy. Front Plant Sci. 2022;12:809828. 35126433 10.3389/fpls.2021.809828 PMC8814108

[46] Zhang J, Zhang D, Liu J, Zhou Y, Cui X, Fan X. DSCONV-GAN: a UAV-BASED model for verticillium wilt disease detection in Chinese cabbage in complex growing environments. Plant Methods. 2024;20(1):186. 10.1186/s13007-024-01303-2. 39696451 10.1186/s13007-024-01303-2 PMC11658099

[47] Fan X, Sun T, Chai X, Zhou J. YOLO-WDNet: A lightweight and accurate model for weeds detection in cotton field. Comput Electron Agric. 2024;225:109317. 10.1016/j.compag.2024.109317.

[48] Zhang Q-Y, Fan K-J, Tian Z, Guo K, Su W-H. High-Precision automated soybean phenotypic feature extraction based on deep learning and computer vision. Plants. 2024;13(18):2613. 39339587 10.3390/plants13182613 PMC11435354

