# Supplementary material for: Advancing radiation-induced mutant screening through high-throughput technology: a preliminary evaluation of mutant screening in Arabidopsis thaliana
Source: Plant Methods. 2025 Apr 15;21:50. doi: 10.1186/s13007-025-01367-8 (PMC11998337; doi:10.1186/s13007-025-01367-8)
Supplement: Supplementary file 1 — Supplementary Material 1 [file 13007_2025_1367_MOESM1_ESM.docx]

| Table S1 |  |  |
| --- | --- | --- |
| Information table of the number of candidate mutant and wild-type plants planted in several experiments | | |
|  |  |  |
| Experiment | The total number of plants | The number of mutants |
| First time | 102 | 8 |
| Second time | 108 | 14 |
| Third time | 139 | 8 |
|  |  |  |

| Table S2 |  |
| --- | --- |
| Candidate mutants numbers and phenotypic information induced by carbon ion beam irradiation and ^60^Co-γ rays | |
|  |  |
| No. of Samples | Phenotypes |
| 7/605 | Dwarfish and tight |
| 116 | Dwarfish, curly leaves |
| 172 | Jagged leaves margin |
| 197 | Tight plant type, dark green leaves |
| 276 | Long growth cycle of 120 days |
| 357 | Dwarfish, polyphyllous |
| 600 | Extremely long petiole |
| 712 | Jagged leaves margin, Slightly curled leaves |
| 828 | Hypertrophied |
| 941 | Dark green, small leaf area |
| 1033 | Clear leaf vein |
| 1267 | Hysteretic bolting |
| 541 | Dwarfish. Branches are perpendicular to stalks. |
