# Supplementary material for: Advancing radiation-induced mutant screening through high-throughput technology: a preliminary evaluation of mutant screening in Arabidopsis thaliana
Source: Plant Methods. 2025 Apr 15;21:50. doi: 10.1186/s13007-025-01367-8 (PMC11998337; doi:10.1186/s13007-025-01367-8)
Supplement: Supplementary file 2 — Supplementary Material 2 [file 13007_2025_1367_MOESM2_ESM.docx]

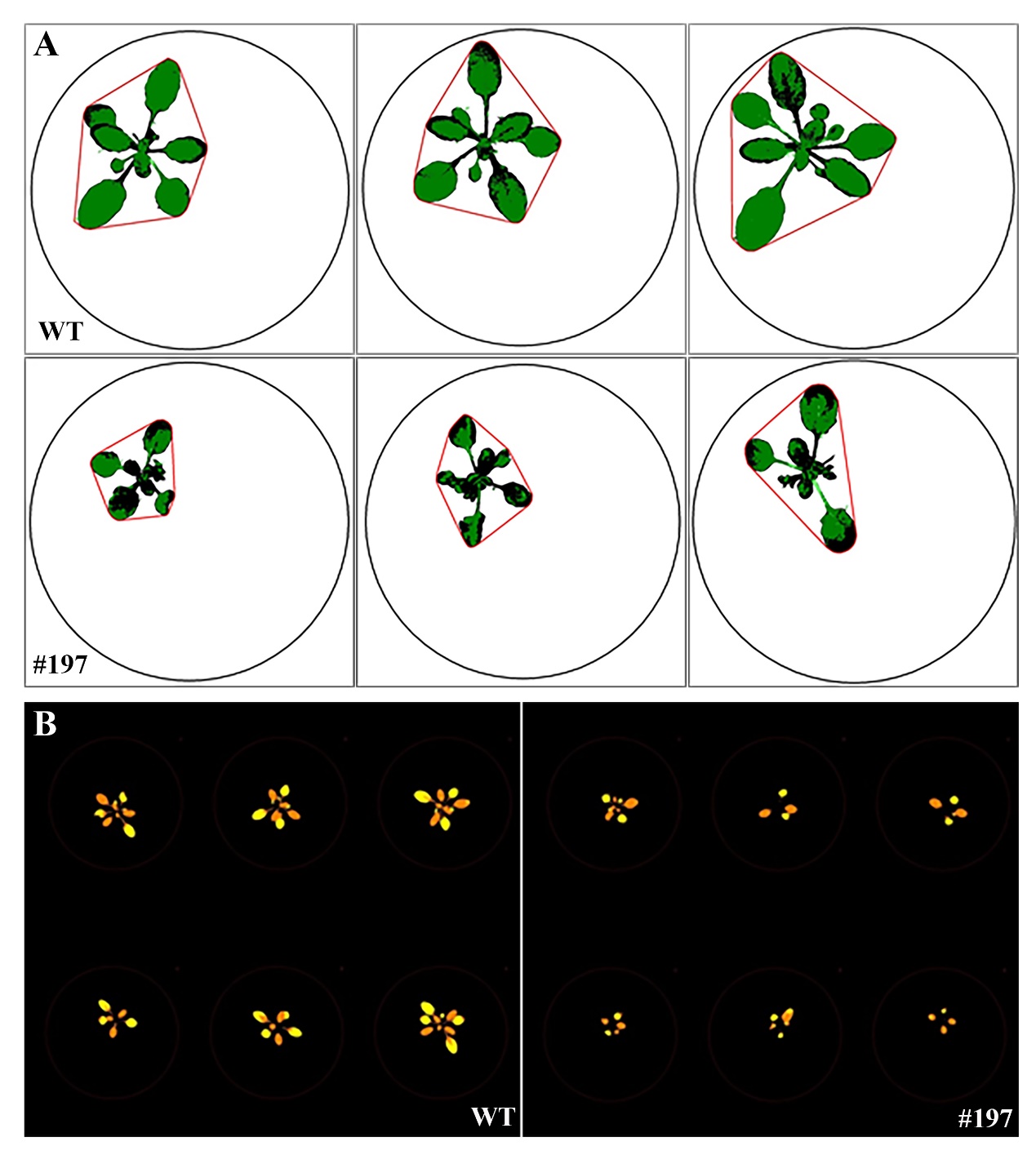


**Fig. S1 Carbon ion beam irradiation-induced phenotype of Arabidopsis #197.** (A) 24 days of post-germination growth under visible light analysis image. (B) 24 days of post-germination growth under fluorescence analysis image.
